# Two new species of *Neotrichoporoides* Girault (Hymenoptera, Eulophidae) from China and a key to Chinese species

**DOI:** 10.3897/zookeys.1023.61580

**Published:** 2021-03-11

**Authors:** Wen-Jian Li, Cheng-De Li

**Affiliations:** 1 School of Forestry, Northeast Forestry University, Harbin, 150040, China Northeast Forestry University Harbin China

**Keywords:** Chalcidoidea, parasitoids, taxonomy, Tetrastichinae

## Abstract

Seven species of *Neotrichoporoides* Girault from China are reviewed, including two new species: *N. basiflavus***sp. nov.**, *N. flavothorax***sp. nov.** and two new country record species: *N. cavigena* Graham, 1991, *N. szelenyii* (Erdös, 1951). New distributional data for *N. mediterraneus* Graham, 1986, *N. nyemitawus* (Rohwer, 1921) and *N. viridimaculatus* (Fullaway, 1955) are provided and a key to Chinese species is given based on females.

## Introduction

The genus *Neotrichoporoides* (Eulophidae: Tetrastichinae) was erected by Girault (1913) with *N. uniguttatus* Girault as type species. Currently the genus contains 73 valid species (Noyes 2019). It is distributed widely and especially diverse in Asia, Africa and Australia (Graham 1987), but only four species were known from China: *N. mediterraneus* Graham, 1986, *N. dubius* (Girault, 1913), *N. nyemitawus* (Rohwer, 1921), and *N. viridimaculatus* (Fullaway, 1955) (Zhu and Huang 2001, 2002; Zhang et al. 2007). Most species of the genus are parasitoids of Diptera in stems of grasses (Graham 1987; LaSalle 1994).

*Neotrichoporoides* can be recognized by the following combination of characteristics (Graham 1987): malar sulcus usually foveate below eyes; antenna of female with four discoid anelli (only three discoid anelli were found in *N. basiflavus* sp. nov.), funicular segments usually elongate; mesosoma with pronotum conical, propodeum usually much longer than dorsellum and strongly reticulate, spiracles small; fore wing with MV 5.5–9.5 × as long as STV, the latter very short; external surface of metacoxae sometimes strongly reticulate; body usually with distinct metallic reflections on dark parts or mainly yellow without metallic reflections.

In the present paper, we add four more species, including two new species and two new country record species to the Chinese fauna. A key to Chinese species is given based on females.

## Materials and methods

Specimens were collected by sweeping, yellow pan trapping and malaise trapping, and were dissected and mounted dorsally in Canada balsam following the method described by Noyes (1982) or glued to triangular cards. Photographs were taken with a digital CCD camera attached to an Olympus BX51 compound microscope and a Aosvi HK-830 microscope. Most measurements were made from slide-mounted specimens using an eye-piece reticle with an Olympus CX21 microscope. In the descriptions below, measurements/ratio in brackets after measurement/ratio ranges refer to the measurement/ratio of the holotype. Terminology follows the Hymenoptera Anatomy Consortium (2020), and the following abbreviations are used:

**F1–4** (flagellomeres 1–4),

**MV** (marginal vein),

**OOL** (minimum distance between lateral ocellus and eye margin),

**OD** (largest diameter of a lateral ocellus),

**POL** (minimum distance between lateral ocelli),

**STV** (stigmal vein),

**SMV** (submarginal vein).

All the specimens listed below were deposited in the insect collections at Northeast Forestry University (**NEFU**), Harbin, China.

## Taxonomy

### Key to the Chinese species of *Neotrichoporoides* Girault (females)

*N. dubius* was excluded from the key because of its insufficient original description.

**Table:** 

1	Mesosoma with combination of yellow and green/black parts (Figs 32, 35)	**2**
–	Mesosoma completely green to black (Figs 33, 34)	**3**
2	Malar sulcus with a subtriangular fovea, extending 0.4–0.5 × the length of malar space (Fig. 11); F1 1.4–1.5 × as long as pedicel (Fig. 12); propodeum completely yellow (Fig. 13)	***N. flavothorax* sp. nov.**
–	Malar sulcus with a small fovea, extending 0.2 × the length of malar space; F1 2.4–2.5 × as long as pedicel; propodeum completely green (Fig. 35)	***N. viridimaculatus* (Fullaway)**
3	Propodeum 2.0–2.5 × as long as dorsellum; midlobe of mesoscutum with two rows of adnotaular setae on each side; externo-dorsal surface of metacoxae with distinct reticulation	**4**
–	Propodeum 1.5 × as long as dorsellum; midlobe of mesoscutum with only one row of adnotaular setae on each side (Fig. 19); externo-dorsal surface of metacoxae without distinct reticulation (Fig. 22)	***N. cavigena* Graham**
4	Antenna with F1 1.4–1.6 × as long as pedicel	**5**
–	Antenna with F1 2.0–2.4 × as long as pedicel	**6**
5	Antennal clava 3.5–3.7 × as long as broad (Fig. 2); lower half of face yellow and basal 1/3 of gaster yellowish (Fig. 31)	***N. basiflavus* sp. nov.**
–	Antennal clava 2.8–3.3 × as long as broad (Fig. 30); face and gaster completely green	***N. mediterraneus* Graham**
6	Antenna with F1 5.2–5.5 × as long as broad (Fig. 29); lower half of face yellow	***N. nyemitawus* (Rohwer)**
–	Antenna with F1 4.0–4.5 × as long as broad (Fig. 24); lower half of face green	***N. szelenyii* (Erdös)**

#### Neotrichoporoides basiflavus sp. nov.

Taxon classificationAnimaliaHymenopteraEulophidae

751DAF94-46CF-5FCE-BA3E-9B3FE7C04518

http://zoobank.org/E14AED3B-638F-4437-8BBB-B1BE84418D5C

Figures 1–6 , 7–10 , 31

##### Type material.

***Holotype***, female [on slide], China, Hainan Province, Haikou City, Hainan University, 27–29.VI.2019, Yu-Ting Jiang, by yellow pan trapping. Deposited in NEFU.

***Paratypes*.** 6 females, 4 males: [2 females and 2 males on slides, 1 male on card], China, Hainan Province, Haikou City, same data as holotype; [2 females on slides, 1 male and 2 females on cards], China, Shandong Province, Qingdao City, Mt. Xiaozhu, 18–20.V.2014, Guo-Hao Zu, Si-Zhu Liu, by yellow pan trapping. All deposited in NEFU.

##### Diagnosis.

**Female.** Body metallic green with lower half of face yellow and basal 1/3 of gaster yellowish; antenna with three discoid anelli, F1 1.4–1.5 × as long as pedicel; midlobe of mesoscutum with two rows of adnotaular setae; fore wing 2.7–2.8 × as long as broad, speculum closed posteriorly; SMV with five setae on dorsal surface. **Male.** Antenna with scape shorter than an eye, reaching above vertex, 4 × as long as broad; ventral plaque 0.63 × as long as scape.

Among the species recorded from China, *N. basiflavus* is similar to *N. mediterraneus* in F1 1.4–1.6 × as long as pedicel, but can be separated from it by the following combination of characteristics: lower half of face yellow and basal 1/3 of gaster yellowish (vs. green); antennal clava 3.5–3.7 × as long as broad (vs. 2.8–3.3 ×); fore wing with speculum closed posteriorly (vs. open posteriorly). The new species is also similar to the extralimital species *N. beonus* Narendran in base of gaster yellow, but can be separated from it by following characteristics: pronotum 0.3–0.5 × as long as mesoscutum (vs. 0.93 ×); fore wing 2.7–2.8× as long as broad (vs. 3.7 ×), SMV with five setae on dorsal surface (vs. six), speculum closed posteriorly (vs. open posteriorly).

##### Description.

**Female. *Body*** length 1.7–2.3 mm (1.8 mm), dark green to green with metallic reflections (Fig. 31). Upper half of face green with metallic reflections, lower half of face yellow, mandibles bronze. Antenna with radicle yellowish, scape mainly yellowish, dark brown along dorsal edge, pedicel with dorsal half dark brown, ventral half yellowish brown, flagellum dark brown. Mesosoma dark green to green with metallic reflections. Wings hyaline, venation yellowish brown. Legs mainly yellow with dorsal half of mesocoxae, and base of metacoxae concolorous with mesosoma, tarsomere 4 of all legs dark brown. Gaster mainly dark green with metallic reflections, with ca. basal 1/3 yellowish, sometimes with a green spot on lateral sides of basal tergite, ovipositor sheaths with third valvula black.

**Figures 1–6.**
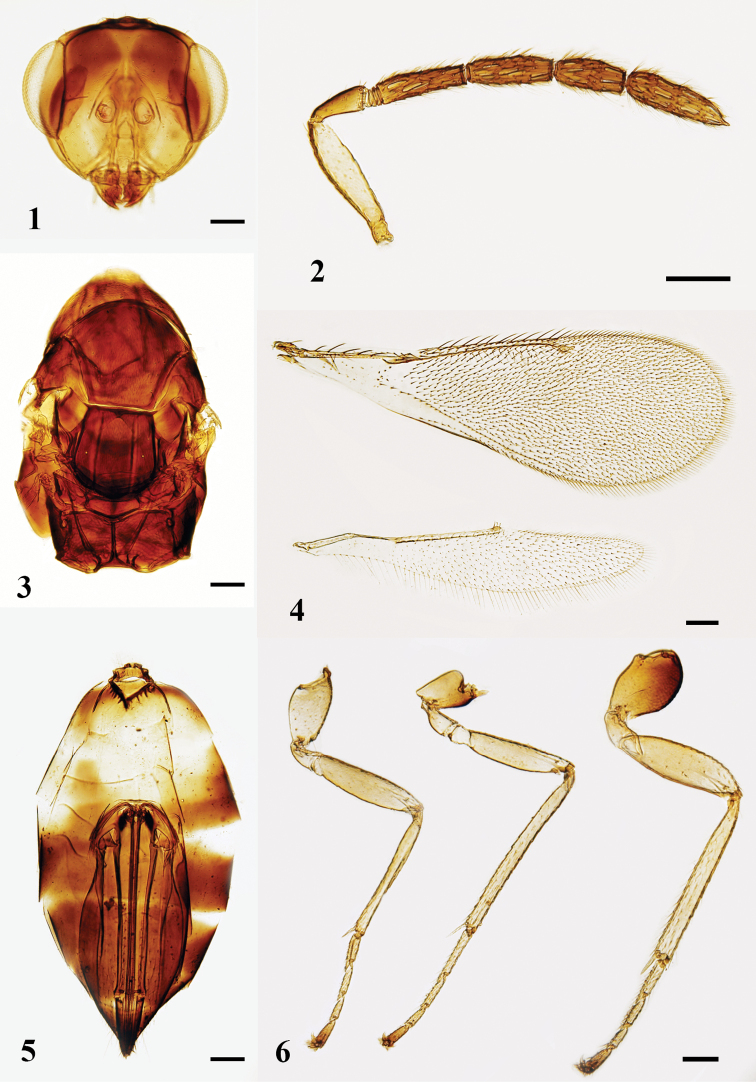
*Neotrichoporoides basiflavus* sp. nov., holotype, female **1** head, frontal view **2** antenna, lateral view **3** mesosoma, dorsal view **4** fore and hind wings, dorsal view **5** metasoma, ventral view **6** legs, lateral view, from left to right: fore, mid, and hind legs. Scale bars: 100 μm.

***Head*** (Fig. 1) in dorsal view, 2.3–2.6 × (2.6 ×) as broad as long, and as broad as mesosoma; POL equal to OOL, OOL 2.8–3.0 × (2.9 ×) OD. Vertex with setae shorter than OD. Eyes separated by 1.2–1.3 × (1.2 ×) their length. Malar space ca. 0.5 × as long as eye, malar sulcus with a subtriangular fovea below eyes, extending ca. 0.5 × the length of malar space; mouth opening 1.5 × as wide as malar space. Clypeus with lower margin bidentate. Mandibles tridentate. Facial depression deep. Torulus with lower margin above the level of ventral margin of eyes. Antenna (Fig. 2) with scape 3.7–4.1 × (3.7 ×) as long as broad, shorter than eye length and not reaching the level of vertex; pedicel 2.3–2.4 × (2.4 ×) as long as broad; with three discoid anelli; F1 3.6–4.3 × (3.7 ×) as long as broad and 1.4–1.5 × (1.4 ×) as long as pedicel, F2 and F3 3.1–3.2 × (3.2 ×) and 2.3–2.6 × (2.3 ×) as long as broad respectively; clava 3.5–3.7 × (3.6 ×) as long as broad, ca. as broad as F3, 0.7 × as long as F2 and F3 combined, sensilla numerous, slender, setae on funicle and clava short and dense.

***Mesosoma*** (Fig. 3) 1.7–1.9 × (1.7 ×) as long as broad. Pronotum subconical, 0.3–0.5 × (0.3 ×) as long as mesoscutum. Midlobe of mesoscutum 1.2 × as broad as long, without median line, with fine reticulation and with two rows of adnotaular setae, four or five setae in outer row and two or three setae in inner row. Scutellum ca. as broad as long; anterior pair of setae distinctly situated before the middle of scutellum, submedian grooves and sublateral grooves distinct, distance between submedian grooves greater than distance between submedian groove and sublateral groove, enclosing a space ca. 2.4 × as long as broad. Reticulation on scutellum similar to that on mesoscutum. Dorsellum 2.5–3.1 × (2.9 ×) as broad as long. Propodeum ca. 2.5 × as long as dorsellum medially; with distinct reticulation, median carina distinct and narrow; spiracles small, circular, separated from metanotum by ca. their own diameter; callus with four or five setae arranged irregularly. Fore wing (Fig. 4) 2.7–2.8 × (2.75 ×) as long as broad, SMV with five setae on dorsal surface; costal cell 0.8 × as long as MV; MV 7.3–8.8 × (8.7 ×) as long as STV with 12–15 setae on its anterior margin; STV short with a long uncus; speculum small, closed posteriorly, subcubital line of setae not reaching to speculum. Hind wing (Fig. 4) 5.0–5.5 × (5.2 ×) as long as broad. Legs (Fig. 6) with metacoxae stout, ca. 1.5 × as long as broad, externo-dorsal surface with distinct reticulation, metafemora 3.3–3.4 × (3.4 ×) as long as broad; spur of metatibia ca. 0.6 × as long as length of metabasitarsus.

***Gaster*** (Fig. 5) lanceolate, slightly depressed dorsally, 2.2–2.5 × (2.5 ×) as long as broad and 1.2–1.5 × (1.4 ×) as long as head and mesosoma combined; petiole transverse; first sternite with a ‘V’ shaped carina and several thin longitudinal carinae; the longest cercal seta 2 × as long as the second longest. Ovipositor originates from ca. basal third of gaster, and is ca. 0.7 × as long as gaster, reaching to, or slightly exserted at, apex of gaster; tip of hypopygium situated at the middle of gaster.

**Figures 7–10.**
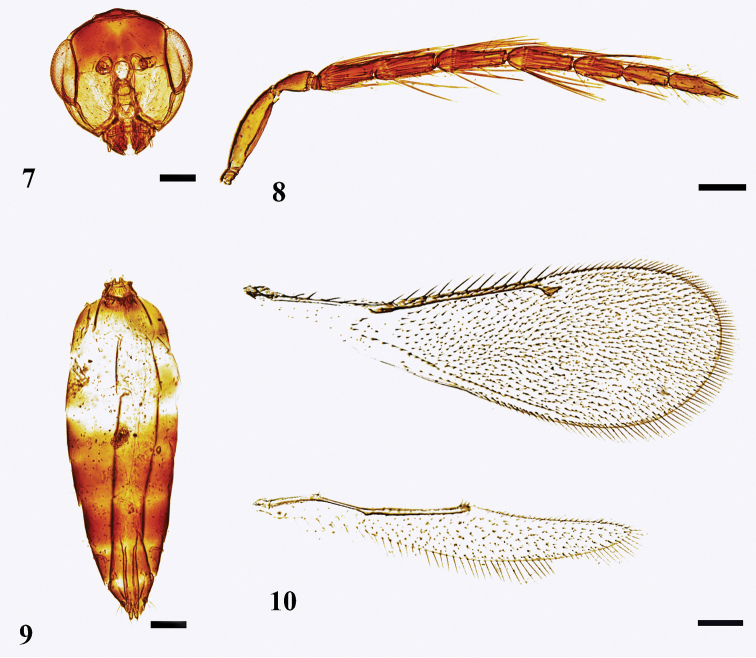
*Neotrichoporoides basiflavus* sp. nov., paratype, male **7** head, frontal view **8** antenna, lateral view **9** metasoma, ventral view **10** fore and hind wings, dorsal view. Scale bars: 100 μm.

**Male.** Similar to female. Head (Fig. 7) as shown. Antenna (Fig. 8) with scape shorter than an eye, reaching above vertex, 4 × as long as broad; ventral plaque 0.60–0.65 × as long as scape; pedicel 1.8 × as long as broad; flagellum slightly broader than pedicel, tapering slightly distally, F1 shortest, 2.1 × as long as broad and 1.6 × as long as pedicel, F2–F4 subequal in length, 3.0 × as long as broad; clava as broad as funicle, 8.5–9.0 × as long as broad, all three segments subequal in length and distinctly separated, terminal spine long, ca. 0.33 × as long as the third segment; funicular segments with whorled long setae, the longest seta on each funicular segment 1.0–1.4 × as long as length of next funicular segment. Fore wing (Fig. 10) with costal cell 0.8 × as long as MV, MV 7.0–8.0 × as long as STV. Gaster (Fig. 9) 2.0–2.5 × as long as broad, 1.0–1.2 × as long as mesosoma; genitalia ca. 2.0 × as long as broad.

##### Host.

Unknown.

##### Distribution.

China (Shandong, Hainan).

##### Etymology.

From the Latin *basis* (base), and *flavus* (yellow), and refers to the yellowish basal part of gaster.

#### Neotrichoporoides flavothorax sp. nov.

Taxon classificationAnimaliaHymenopteraEulophidae

DD0C9833-AAF7-554F-8E0B-09F95630ED86

http://zoobank.org/C6AC84DD-B3CB-4002-A8B5-6D0CA984E2B7

Figures 11–16 , 32

##### Type material.

***Holotype***, female [on slide], China, Shandong Province, Qingdao City, Mt. Xiaozhu, 18–20.V.2014, Guo-Hao Zu, Si-Zhu Liu, by yellow pan trapping. Deposited in NEFU.

***Paratypes*.** 2 females: [1 female on slide], same data as holotype; [1 female on slide], China, Hainan Province, Wan Ning City, Shuangxi Village, 17–19.IV.2019, Yu-Ting Jiang, by yellow pan trapping. All deposited in NEFU.

##### Diagnosis.

**Female.** Body mainly yellow with green or black markings (Fig. 32); F1 4.2 × as long as broad, 1.4–1.5 × as long as pedicel; mid lobe of mesoscutum with three adnotaular setae in one row; propodeum 2.0–2.3 × as long as dorsellum; fore wing with MV 9.5 × as long as STV, speculum closed posteriorly.

Among the species recorded from China, *N. flavothorax* is similar to *N. viridimaculatus* (Fullaway) in having similar combination of yellow and green/black parts on mesosoma, but can be separated from *N. viridimaculatus* by the following characteristics: propodeum completely yellow (vs. completely green); malar sulcus with a subtriangular fovea, extending 0.4–0.5 × the length of malar space (vs. small, 0.2 ×); F1 1.4–1.5 × as long as pedicel (vs. 2.4–2.5 ×). The new species is also similar to the extralimital species *N. dispersus* Graham in having similar combination of yellow and green/black parts on mesosoma, but can be separated by the following characteristics: propodeum completely yellow (vs. partly green); F1 1.4–1.5 × as long as pedicel (vs. 2.4–2.5 ×).

##### Description.

**Female. *Body*** length 1.9–2.3 mm (1.9 mm). Head with upper half of face and posterior upper part of gena green with metallic reflections, lower half of face yellow; vertex with subtriangular ocelli area and occiput black, mandibles bronze; antenna with radicle, scape and pedicel yellow, flagellum brown. Mesosoma mainly yellow (Fig. 32), with pronotum, anterior middle part of mid lobe of mesoscutum black, scutellum green with metallic reflections; legs mainly yellow except tarsomere 4 of all legs dark brown; wings hyaline, venation yellowish brown. Gaster mainly dark brown with basal 1/3 yellow and a yellow spot on the terminal part of gaster, ovipositor sheaths with 1/3 valvula black.

**Figures 11–16.**
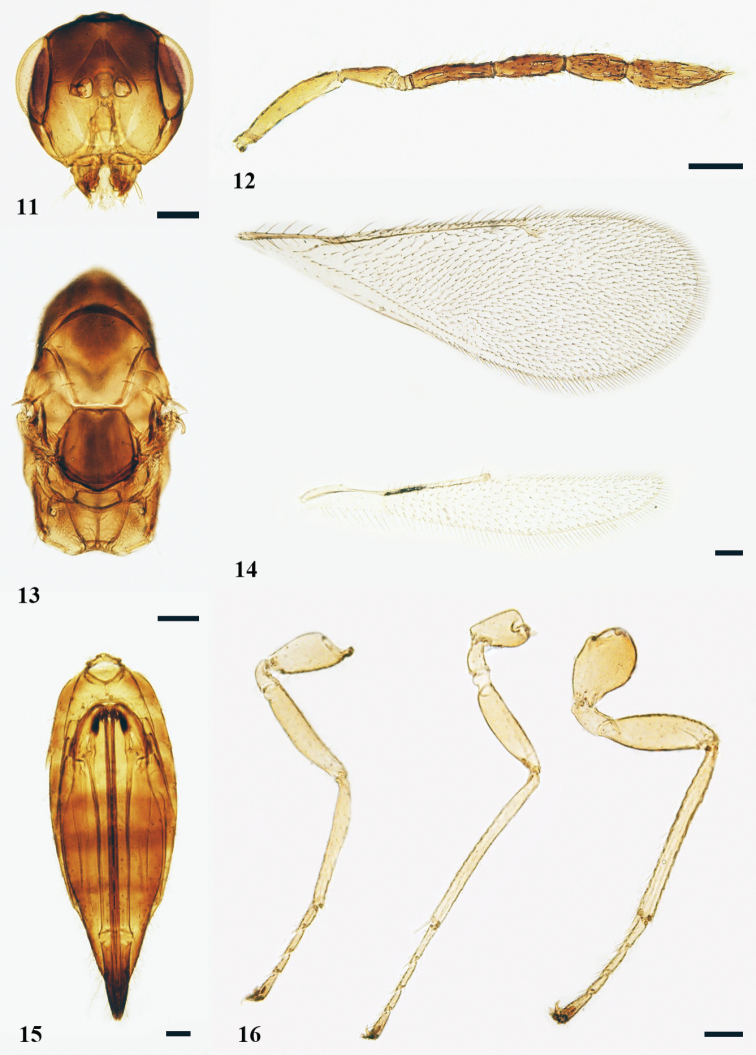
*Neotrichoporoides flavothorax* sp. nov., holotype, female **11** head, frontal view **12** antenna, lateral view **13** mesosoma, dorsal view **14** fore and hind wings, dorsal view **15** metasoma, ventral view **16** legs, lateral view, from left to right: fore, mid, and hind legs. Scale bars: 100 μm.

***Head*** (Fig. 11) in dorsal view, 2.3 × as broad as long, 1.0–1.1 × as broad as mesosoma. POL 1.2–1.3 × (1.3 ×) OOL, OOL 2.5 × OD. Eyes separated by 1.2 × their length. Malar space ca. 0.6 × as long as eye, malar sulcus with a subtriangular fovea below eyes, extending 0.4–0.5 × (0.4 ×) the length of malar space; mouth opening 1.5 × as wide as malar space. Clypeus with lower margin bidentate. Mandibles tridentate. Facial depression shallow. Torulus with lower margins above the level of ventral margin of eyes. Antenna (Fig. 12) with scape 4.5–5.0 × (5.0 ×) as long as broad, slightly shorter than eye length and reaching above the level of vertex; pedicel 2.6× as long as broad; with four discoid anelli; F1 4.2 × as long as broad, 1.4–1.5 × (1.5 ×) as long as pedicel, F2 and F3 3.2–3.3 × (3.3 ×) and 2.3–2.4 × (2.3 ×) as long as broad respectively; clava 3.8–4.0 × (3.8 ×) as long as broad, 0.8 × as long as F2 and F3 combined, indistinctly segmented and pointed at apex, sensilla numerous, slender; setae on flagellum short and dense.

***Mesosoma*** (Fig. 13) 1.9 × as long as broad. Pronotum subconical, 0.3–0.4 × (0.4 ×) as long as mesoscutum. Mid lobe of mesoscutum ca. as broad as long, without median line, with extremely fine reticulation and three adnotaular setae in one row. Scutellum as broad as long; anterior pair of setae situated distinctly before the middle of scutellum, submedian grooves superficial and sublateral grooves distinct, distance between submedian grooves greater than distance between submedian groove and sublateral groove, enclosing a space ca. 2.2 × as long as broad. Reticulation on scutellum similar to that on mesoscutum. Dorsellum ca. 2.5 × as broad as long, without reticulation, posterior edge slightly curved. Propodeum 2.0–2.3 × as long as dorsellum medially, with distinct reticulation, median carina distinct and narrow; spiracles small, circular, separated from anterior margin of propodeum by ca. their own diameter; callus with three setae. Fore wing (Fig. 14) 2.8 × as long as broad, SMV with five setae on dorsal surface; costal cell 0.62 × as long as MV; MV 9.5 × as long as STV; STV with a long uncus; speculum small, closed posteriorly. Hind wing 6.2 × as long as broad, pointed. Legs (Fig. 16) with metacoxae stout, ca. 1.4 × as long as broad, externo-dorsal surface with fine reticulation, metafemora 3.6 × as long as broad; spur of metatibia 0.7 × as long as length of metabasitarsus.

***Gaster*** (Fig. 15) lanceolate, not depressed dorsally, 3.0 × as long as broad and 1.4 × as long as head and mesosoma combined; petiole transverse; the longest cercal seta 2 × as long as the second longest. Ovipositor ca. 0.9 × as long as gaster and slightly exserted at apex of gaster; tip of hypopygium situated at ca. basal 1/3 of gaster.

**Male.** Unknown.

##### Host.

Unknown.

##### Distribution.

China (Shandong, Hainan).

##### Etymology.

From the Latin *flavus* (yellow), and refers to the mainly yellow thorax of the species.

#### Neotrichoporoides cavigena

Taxon classificationAnimaliaHymenopteraEulophidae

Graham, 1987

41C31086-9584-5356-92E0-DECB04CB6C51

Figures 17–22

Neotrichoporoides cavigena Graham, 1987: 70.

##### Material examined.

2 females: [2 females on slides], China, Beijing, Mt. Baihua, 1.V.2012, Guo-Hao Zu, Jiang Liu, by sweeping. All deposited in NEFU.

##### Diagnosis.

**Figures 17–22.**
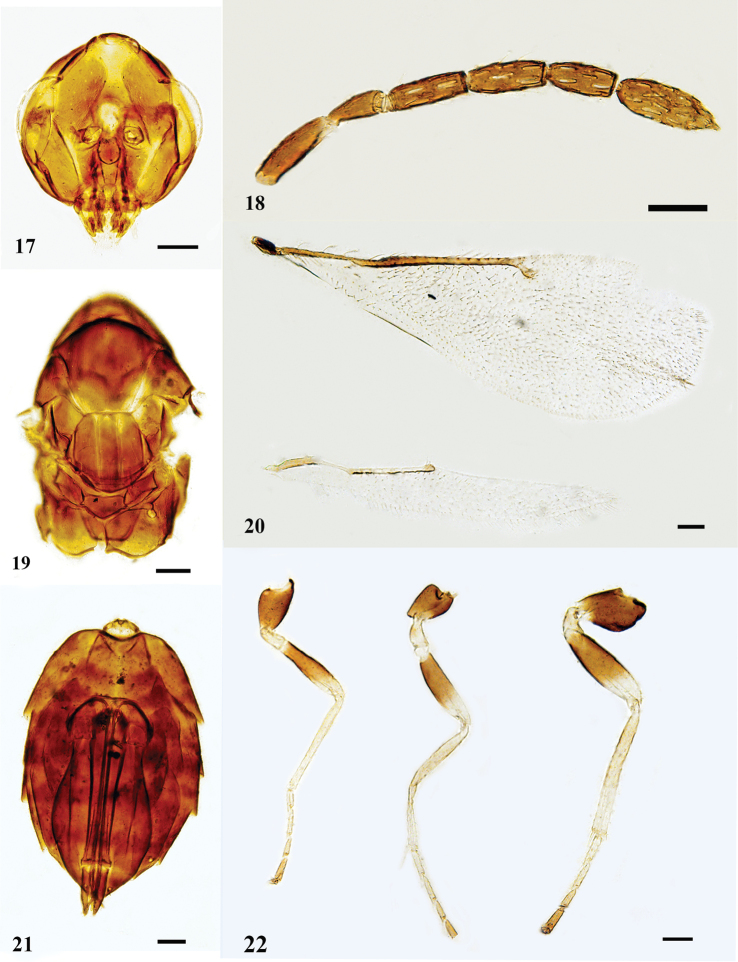
*N. cavigena*, female **17** head, frontal view **18** antenna, lateral view **19** mesosoma, dorsal view **20** fore and hind wings, dorsal view **21** metasoma, ventral view **22** legs, lateral view, from left to right: fore, mid, and hind legs. Scale bars: 100 μm.

**Female.** Head (Fig. 17) with malar fovea large and deep, extending ca. half the length of malar space; antenna (Fig. 18) with scape ca. 3.3 × as long as broad, shorter than an eye, not reaching above the level of vertex; pedicel 2.35 × as long as broad; F1–F3: 3.0 ×, 2.8 ×, 2.4 × as long as broad respectively; clava ca. 3.0 × as long as broad, indistinctly segmented. Midlobe of mesoscutum (Fig. 19) with four adnotaular setae in one row; scutellum with submedian grooves distinct, distance between submedian grooves subequal to the distance between submedian groove and sublateral groove, enclosing a space ca. 3.5 × as long as broad; propodeum medially 1.5 × as long as dorsellum. Wings (Fig. 20) and legs (Fig. 22) as shown in figures. Gaster (Fig. 21) ca. 1.8 × as long as broad. **Male.** Unknown for Chinese material.

##### Host.

Unknown.

##### Distribution.

China (Beijing) [new record], Bulgaria, France, Czech Republic (Graham 1987), Slovakia (Kalina 1989), Russia (Yegorenkova and Kostjukov 2006), Turkey (Sakaltaş and Gençer 2005).

##### Comments.

This species can be distinguished by the narrow space, ca. 3.5 × as long as broad, enclosed by submedian grooves on the scutellum. For a more detailed description, see Graham (1987).

#### Neotrichoporoides szelenyii

Taxon classificationAnimaliaHymenopteraEulophidae

(Erdös, 1951)

BFCB4339-F638-5D31-9F25-1E65F8C772D2

Figures 23–28 , 33

Geniocerus szelenyii Erdös, 1951: 232. Lectotype designated by Graham 1987: 69.
Aprostocetus szelenyii : Graham, 1961: 50.
Tetrastichus szelenyi : Bouček, 1965: 212 (misspelling).
Tetrastichus szelenyii : Domenichini, 1966b: 50.
Neotrichoporoides szelenyii : Graham, 1987: 68.
Neotrichoporoides szelynii : Yefremova, 2008: 358 (misspelling).

##### Material examined.

8 females: [2 females on slides], China, Hainan Province, Haikou City, Hainan University, 27–29.VI.2019, Yu-Ting Jiang, by yellow pan trapping; [2 females on slides], Hainan Province, Chengmai County, Jinjiang Town, 24–26.IV.2019, Yu-Ting Jiang, by yellow pan trapping; [4 females on cards], Shanghai City, Songjiang District, Yexie Town, 11–20.IX.2011, Zhen Yang, by malaise trapping. All deposited in NEFU.

##### Diagnosis.

**Figures 23–28.**
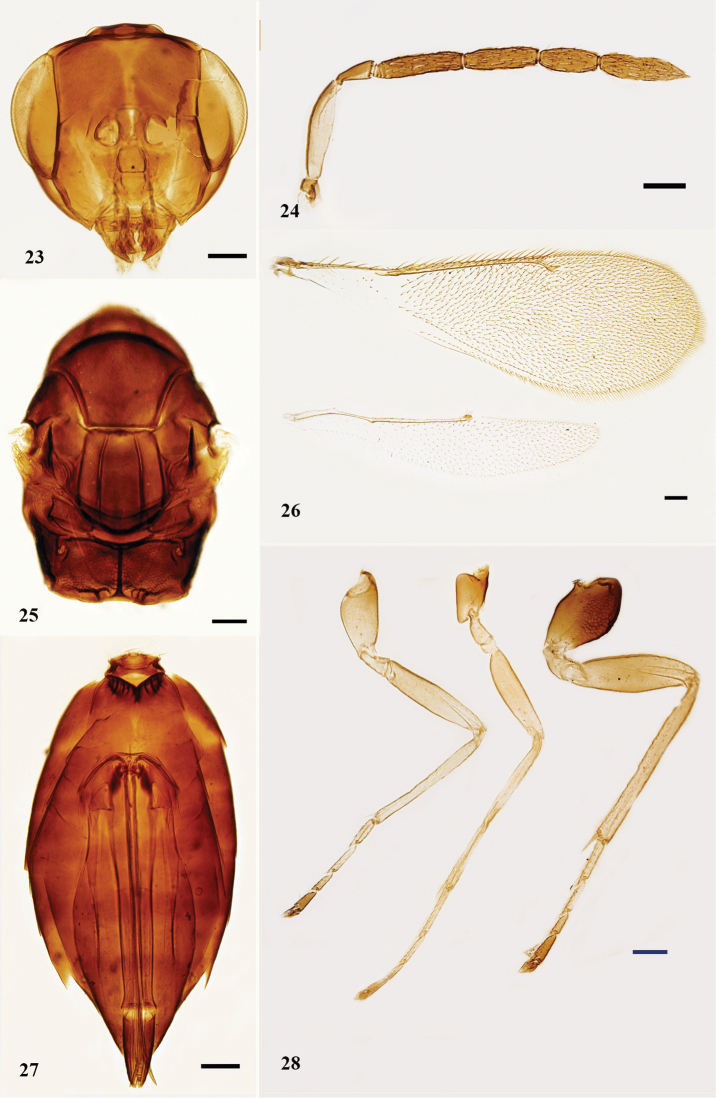
*N. szelenyii*, female **23** head, frontal view **24** antenna, lateral view **25** mesosoma, dorsal view **26** fore and hind wings, dorsal view **27** metasoma, ventral view **28** legs, lateral view, from left to right: fore, mid, and hind legs. Scale bars: 100 μm.

**Female.** Antenna (Fig. 24) with scape 0.9–1.0 × as long as an eye, F1 4.0–4.5 × as long as broad, ca. 2 × as long as pedicel and 0.9 × as long as clava; F2 2.8–3.3 × as long as broad; F3 2.0–2.6 × as long as broad; clava 3.6–4.0 × as long as broad. Propodeum (Fig. 25) medially 2.5 × as long as dorsellum. Fore wing (Fig. 26) 2.7–2.8 × as long as broad, SMV with five to seven setae on dorsal surface; costal cell 0.8 × as long as MV, MV 8.0–8.8 × as long as STV; speculum open posteriorly. Gaster (Fig. 27) 2.3–2.5 × as long as broad and 1.1–1.3 × as long as head and mesosoma combined. Head (Fig. 23) and legs (Fig. 28) as shown in figures. **Male.** Unknown for Chinese material.

##### Host.

Unknown.

##### Distribution.

China (Hainan, Shanghai) [new record], Azerbaijan, Hungary, Portugal (Graham 1987), Italy, Greece, Bulgaria (Boyadzhiev 1999), Czechoslovakia, Moldova (Bouček 1965), Iran (Hesami et al. 2010), Romania (Hansson 2016), Turkey (Sakaltaş and Gençer 2005), Saudi Arabia (OILB 1971), United Arab Emirates (Yefremova 2008).

##### Comments.

This species is similar to *N. mediterraneus*, but can be distinguished using characters in couplet 6 in the key.

#### Neotrichoporoides mediterraneus

Taxon classificationAnimaliaHymenopteraEulophidae

Graham, 1986

F7F4F997-77FE-50AA-B837-71658B4533AD

Figure 30

Neotrichoporoides mediterraneus Graham, 1986: 6.

##### Material examined.

2 females: [1 female on slide], Henan Province, Xinyang City, Mt. Wusheling, 7.VIII.2015, Hui Geng, Yan Gao, by sweeping; [1 female on slide], Guangxi Province, Fangchenggang City, Mt. Shiwandashan, 25.VII.2019, Jun Wu, Jun-Jie Fan, by sweeping. All deposited in NEFU.

##### Diagnosis.

**Female.** Antenna (Fig. 30) with scape 0.8–0.9 × as long as an eye, F1 3.0–4.0 × as long as broad, 1.4–1.6 × as long as pedicel; F2 3.0–3.7 × as long as broad; F3 2.0–2.6 × as long as broad; clava 2.8–3.3 × as long as broad. Fore wing 2.7–2.8 × as long as broad, SMV with five setae on dorsal surface, MV 8.0–9.3 × as long as STV; speculum open posteriorly. Gaster 2.4 × as long as broad and 1.2 × as long as head and mesosoma combined. **Male.** Unknown for Chinese material.

**Figures 29, 30.**
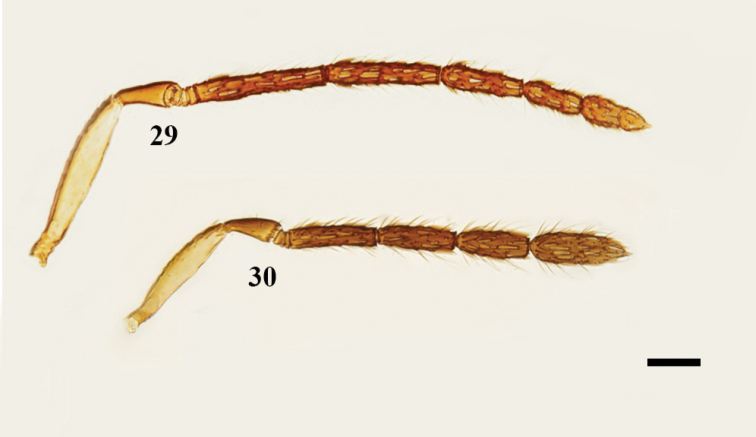
Females **29***N. nyemitawus*, antenna, lateral view **30***N. mediterraneus*, antenna, lateral view. Scale bars: 100 μm.

##### Host.

Unknown.

##### Distribution.

China (Guangxi (Zhu and Huang 2002), Henan [New record]), Bulgaria, Czech Republic, Slovakia (Boyadzhiev 1999), Spain, France, Italy (Graham 1987), Romania (Hansson 2016), Russia (Yegorenkova and Kostjukov 2006), Turkey (Sakaltaş and Gençer 2005), India (Graham 1987), Australia (Bouček 1988), Canary Islands, Madeira (Graham 1987).

##### Comments.

According to Graham (1986), *N. mediterraneus* is quite similar to *N. szelenyii*. For a more detailed description, see Graham (1986).

#### Neotrichoporoides nyemitawus

Taxon classificationAnimaliaHymenopteraEulophidae

(Rohwer, 1921)

13E18107-B98E-512B-836F-397189EDA1B0

Figures 29 , 34

Tetrastichus nyemitawus Rohwer, 1921: 131.
Tetrastichus agarwali Shafee, Fatma & Kishore, 1984: 393. [Synonymized by Hayat and Shahi 2004: 308].
Neotrichoporoides nyemitawus : Graham, 1987: 68.

##### Material examined.

3 females: [1 female on slide], Henan Province, Xinyang City, Mt. Wusheling, 7.VIII.2015, Hui Geng, Yan Gao, by sweeping; [1 female on slide], Zhejiang Province, Jinhua City, Xishan Village, 25–27.VI.2019, by yellow pan trapping; [1 female on card], Yunnan Province, Tengchong City, Guanpojiao, Xiang-Xiang Jin, Guo-Hao Zu, Chao Zhang, by sweeping. All deposited in NEFU.

##### Diagnosis.

**Female.** Antenna (Fig. 29) with scape ca. as long as an eye, reaching well above the level of vertex; F1 5.2–5.5 × as long as broad, 2.2–2.4 × as long as pedicel; F2 4.1 × as long as broad; F3 3.0–3.1 × as long as broad; clava 4.2–4.7 × as long as broad, distinctly segmented. Fore wing 3.0 × as long as broad, SMV with five to seven setae on dorsal surface, MV 8.0–9.3 × as long as STV. Gaster (Fig. 34) 2.6–3.0 × as long as broad and 1.2–1.3 × as long as head and mesosoma combined. **Male.** Unknown for Chinese material.

##### Hosts.

Unknown from China. Non-Chinese records include *Atherigona naqvii* (Husain & Khan, 1986), *A. conigera*, *A. soccata* (Graham, 1987), *A. hyalinipennis* (Sileshi, 1997), *A. varia* (Raodeo, Tikar & Chundurwar, 1972) (Diptera: Anthomyiidae).

##### Distribution.

China (Gansu, Jiangsu (Zhang et al. 2007), Zhejiang (Zhu and Huang 2001), Guangxi (Zhu and Huang 2002), Henan, Yunnan [new records]), Thailand, India, Kenya (Graham 1987), Ethiopia (Sileshi 1997), Burkina Faso (Zongo, Vincent and Stewart 1993).

##### Comments.

This species can be distinguished by its distinctly segmented clava that is 4.2–4.7 × as long as broad, and the yellow lower half of face.

#### Neotrichoporoides viridimaculatus

Taxon classificationAnimaliaHymenopteraEulophidae

(Fullaway, 1955)

63E989B0-361A-5275-B81C-070EB0D72CCB

Figure 35

Burksia viridimaculata Fullaway, 1955: 410.
Tetrastichus viridimaculatus : Domenichini, 1966a: 140.
Tetrastichus bicolor Saraswat, 1975: 2. [Synonymised by Hayat and Shahi 2004: 309].
Tetrastichus saraswati Husain & Khan, 1986: 242. [Synonymised by Hayat and Shahi 2004: 309].
Neotrichoporoides viridimaculatus : Graham, 1987: 67.

##### Material examined.

6 females, 2 males: [1 female on slide], Henan Province, Xinyang City, Mt. Wusheling, 8.VIII.2015, Hui Geng, Yan Gao, by sweeping; [1 female on card], Guangxi Province, Fangchenggang City, Mt. Shiwandashan, 25.VII.2019, Jun Wu, Jun-Jie Fan, by sweeping; [4 females, 2 males on cards], Shanghai City, Songjiang District, Yexie Town, 11–20.IX.2011, Zhen Yang, by malaise trapping. All deposited in NEFU.

##### Diagnosis.

**Female.** Malar sulcus with a small fovea, extending 0.2 × the length of malar space; antenna with scape ca. as long as an eye; F1 2.4–2.5 × as long as pedicel; scutellum without submedian grooves; propodeum medially 1.5–2.0 × as long as dorsellum; body (Fig. 35) with characteristic green markings on midlobe of mesoscutum and scutellum which form broad longitudinal stripes, propodeum completely green. **Male.** Scutellum without submedian grooves.

**Figures 31–35.**
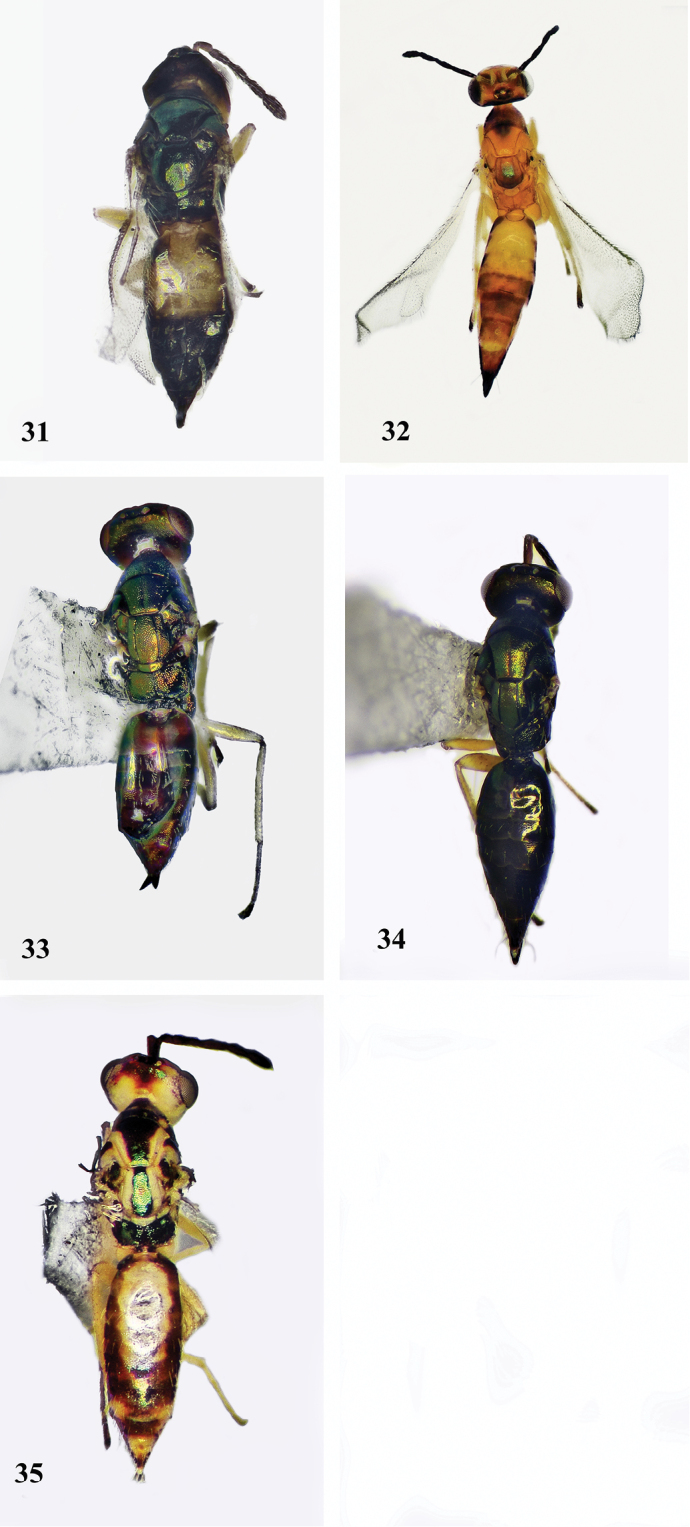
Females, dorsal view **31***Neotrichoporoides basiflavus* sp. nov. **32***Neotrichoporoides flavothorax* sp. nov. **33***N. szelenyii***34***N. nyemitawus***35***N. viridimaculatus*

##### Host.

Unknown.

##### Distribution.

China (Gansu (Zhang et al. 2007), Zhejiang (Zhu and Huang 2001), Guangxi, Henan, Shanghai [New records]), Bulgaria, France, Hungary, Czechoslovakia, Italy, Madeira, Portugal (Graham 1987), Sweden (Hedqvist 2003), Turkey (Sakaltaş and Gençer 2005), Russia (Yegorenkova and Kostjukov 2006), India (Narendran et al. 2006), South Africa (Yegorenkova and Yefremova 2010), USA (LaSalle 1994), Hawaii (Graham 1987), Cuba (De Santis 1979), Bermuda (De Santis and Fidalgo 1994), Argentina (Graham 1987), Colombia (Domenichini 1966b).

##### Comments.

This species is similar to *Neotrichoporoides flavothorax* sp. nov., but can be distinguished using characters in couplet 2 in the key.

## Supplementary Material

XML Treatment for Neotrichoporoides basiflavus

XML Treatment for Neotrichoporoides flavothorax

XML Treatment for Neotrichoporoides cavigena

XML Treatment for Neotrichoporoides szelenyii

XML Treatment for Neotrichoporoides mediterraneus

XML Treatment for Neotrichoporoides nyemitawus

XML Treatment for Neotrichoporoides viridimaculatus
